# Environment-dependent degradation pathways of pure zinc induced by acid–alkali surface chemistry

**DOI:** 10.1039/d6ra00345a

**Published:** 2026-04-21

**Authors:** Abdalla Abdal-hay, Enmao Xiang, Yahia Ali, Jiaqi Xu, Yu Kyoung Kim

**Affiliations:** a Centre for Orofacial Regeneration, Reconstruction and Rehabilitation (COR3), School of Dentistry, The University of Queensland Brisbane 4006 Australia abdalla.ali@uq.edu.au; b Centre for Advanced Materials Processing and Manufacturing (AMPAM), School of Mechanical and Mining Engineering, The University of Queensland Brisbane Qld 4072 Australia; c The Conversationalist Club & Department of Dental Digitalization, School of Stomatology, Shandong First Medical University Jinan 250117 Shandong China; d School of Dentistry, Jeonbuk National University Jeon Ju South Korea; e Charles Perkins Centre, The University of Sydney Camperdown NSW 2006 Australia; f Sydney Dental School, Faculty of Medicine and Health, The University of Sydney Camperdown NSW 2006 Australia

## Abstract

Pure zinc (Zn) is a promising candidate for biodegradable metallic implants; however, its degradation behaviour in physiological environments is highly sensitive to interfacial chemistry, often resulting in non-uniform and environment-dependent corrosion responses that complicate controlled tissue regeneration. This study introduces a simple two-step chemical modification, consisting of acid etching followed by alkaline treatment, to tailor the early-stage degradation behaviour of pure Zn. The degradation behaviour of untreated and treated Zn was systematically evaluated in Hanks' balanced salt (HBSS), 0.85 wt% NaCl and 10 mM Tris–HCl solutions at 37 °C. Electrochemical measurements, immersion tests and surface characterisation studies were conducted. The results show that the combined acid–alkali treatment did not merely accelerate degradation, but altered the dominant degradation pathways in a strongly media-dependent manner. In HBSS, chemically treated Zn promoted the formation of Ca/P-rich surface layers, which was associated with more moderate and spatially homogeneous degradation behaviour, whereas chloride- and buffer-dominated environments favoured film destabilisation or enhanced dissolution. These findings demonstrate that surface chemistry can be used to control environment-specific degradation pathways of pure Zn without alloying, providing a new design strategy for biodegradable Zn-based implants.

## Abstract 2

A simple acid–alkali surface treatment tailors pure zinc's degradation in a medium-specific way, enabling more uniform and controlled biodegradation in physiological environments without alloying.

## Graphical abstract

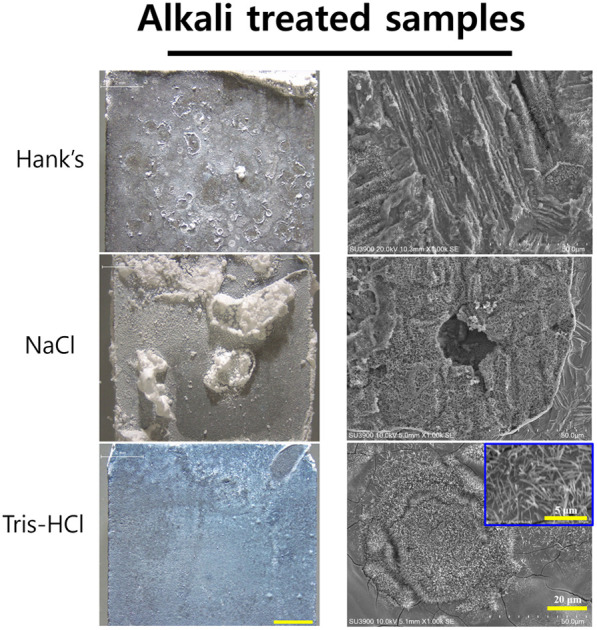

## 1. Introduction

The use of bone implants has shown remarkable growth over the last decades, driven by several factors, including the desire of patients to maintain the same level of activity and quality of life.^1^ Bone cancer is the second most common cause of death in children, adolescents, and young adults after brain tumors. As a result, the demand for high-performance implantable biomaterials that can address unique challenges in trauma, orthopaedics, dentistry, and wound care is steadily increasing.

To mitigate the long-term side effects associated with permanent implants, biodegradable metallic implants, including magnesium (Mg), iron (Fe), and zinc (Zn) based alloys have attracted the attention of the scientific community.^2^ One of their key features is controlled degradation to accommodate bone healing and growth.^3^

The latest generation of biodegradable metal implants are revolutionary. Not only do they promote the formation of new bone, but they also eliminate the stress shielding – typical of current implants – and the permanent physical irritation to the tissue, making implant removal surgery unnecessary.^4,5^

Amongst the latest generation of biodegradable metals, Zn and its alloys have received the most attention for degradable implants for two reasons. One, their mechanical properties can be slightly tailored to approach those of cortical bone. Second, they possess intrinsic biodegradability in physiological environments together with generally acceptable biocompatibility.^6^ Nevertheless, the *in vivo* degradation rate of current Zn-based alloys often remains below the clinically desirable threshold, meaning that implants degradation is slower than the tissue healing. This has significantly limited clinical application of these alloys.^7^ Therefore, controlling the degradation characteristics of Zn and its alloys to match the tissue growth rate is a hot research topic nowadays.

Many promising Zn alloys have been developed, and their microstructures, mechanical properties, degradation behaviour, ion release characteristics, and both *in vitro* and *in vivo* biocompatibility have been extensively investigated.^8,9^ While alloying strategies effectively tailor strength and average corrosion rates, pure and alloyed Zn remain susceptible to localized and pitting corrosion in chloride-containing physiological environments. Such heterogeneous degradation can lead to asymmetric loss of cross-sectional integrity, premature mechanical failure, and transient zinc ion bursts that may compromise local cellular responses. Therefore, strategies capable of regulating and homogenizing Zn degradation—without sacrificing mechanical integrity—are critical for reliable clinical translation.

Surface engineering provides a targeted approach by modifying the implant-electrolyte interface where corrosion reactions initiate. Coatings such as calcium phosphate, plasma electrolytic oxidation (PEO), and atomic layer deposition (ALD) have demonstrated the ability to modulate corrosion kinetics, ion release, cytocompatibility, and hemocompatibility.^3,4^ However, many of these approaches rely on hydrothermal processing, electrochemical deposition, or vacuum-assisted techniques requiring specialized equipment and strict processing control,^10–12^ raising concerns regarding scalability and manufacturing cost.

Beyond biomedical surface coatings, electrochemical corrosion studies have demonstrated that zinc degradation in chloride media is highly sensitive to interfacial chemistry and adsorbed organic species. For example, alkyl-phosphonic acids form chemisorbed zinc-phosphonate complexes on oxide-hydroxide surface layers, producing hydrophobic films that suppress chloride-induced depassivation and significantly enhance corrosion resistance in NaCl solutions.^6,10^ Similarly, alkyl-malonate derivatives exhibit strong chemisorption on oxidized zinc surfaces (–Δ*G* ads up to 76.7 kJ mol^−1^), forming nanoscale adsorption films that provide complete protection in 0.01 M NaCl under natural aeration for extended durations.^9,12^ These studies demonstrate that even nanometric organic surface layers can fundamentally modify anodic and cathodic reactions on zinc by altering adsorption equilibria, interfacial water structure, and chloride accessibility.

In contrast to hydrophobic barrier approaches, our recent work introduced a simple sequential acid–alkali chemical treatment for pure Zn.^13^ Acid etching (H_2_SO_4_/HCl/H_2_O) followed by NaOH immersion generated a hydroxyl-rich and microstructured surface that significantly accelerated heterogeneous nucleation of calcium phosphate (CaP) in HBSS. The treated surfaces promoted rapid CaP deposition and improved osteoblast attachment and proliferation, demonstrating enhanced osteogenic bioactivity.^13^ Unlike phosphonate-based passivation strategies that suppress degradation, this acid–alkali modification was designed to promote controlled surface reactivity and biomineralization.

However, while enhanced CaP formation was demonstrated under a single simulated physiological condition, it remains unclear whether acid–alkali treatment merely accelerates mineral deposition or fundamentally alters the degradation mechanisms of Zn across chemically distinct environments. Prior corrosion studies clearly show that zinc behaviour differs substantially between chloride-dominated electrolytes and buffered systems containing complexing or precipitating species.^10–12^ Chloride ions promote localized depassivation and pitting, whereas Ca/P-containing media favor formation of zinc phosphate layers that can either stabilize or segment corrosion fronts depending on local supersaturation and transport conditions.^11,12^

A critical limitation of the current Zn biomaterials literature is the frequent reliance on single-medium degradation testing, implicitly assuming transferable corrosion behaviour. Yet, as demonstrated in electrochemical corrosion research and *in vivo* studies,^12^ surface films such as ZnO, Zn(OH)_2_, zinc phosphate, or organic adsorption layers evolve differently depending on electrolyte composition and mass transport conditions. Consequently, degradation morphology, ion release kinetics, and biological responses cannot be inferred from a single testing environment.

Therefore, the present study systematically investigates the degradation behaviour of acid–alkali treated pure Zn in Ca/P-containing, chloride-dominated, and buffer-controlled media. By correlating surface chemistry, corrosion morphology, and degradation kinetics across chemically distinct environments, we elucidate whether this simple and scalable chemical treatment modifies only surface bioactivity or fundamentally reshapes Zn degradation pathways in an environment-dependent manner. This work bridges corrosion science and biodegradable implant design, providing mechanistic insight necessary for tailoring Zn surface states according to specific physiological applications.

## 2. Materials and methods

### 2.1 Preparation and chemical treatment of Zn specimens

Zn samples were cut from commercially available Zn sheets (99.5 wt% purity, Nilaco, Tokyo, Japan). According to the supplier specifications, the main trace impurities include Pb (<0.02 wt%), Fe (<0.01 wt%), Cu (<0.01 wt%) and Cd (<0.005 wt%). These impurity levels are typical for commercially available high-purity Zn and are not expected to significantly affect the corrosion behaviour under the present experimental conditions 10 mm quare were cut pure Zn sheet. Each sample measured 1 mm in thickness. Prior to the surface treatment, all samples were mechanically ground and polished using successive grades of SiC emery papers (grit 320–1200) and diamond polishing suspension (Struers, Milton, Australia). They were then thoroughly rinsed with Milli-Q ultrapure water and dried in ambient air.

For chemical treatment, samples were first etched in a 1 : 1 : 9 (H_2_SO_4_ : HCl : H_2_O) acid solution at room temperature for 0.5 h to remove the native oxide layer and increase the surface roughness. The samples were then immersed in 200 mL of 5.0 M sodium hydroxide (NaOH) solution at 37 °C for 24 hours and Shaked at 80. For clarity, the group referred to throughout this manuscript as ‘alkali-treated’ corresponds to the sequential acid-etching followed by NaOH treatment, rather than an alkali-only treatment, to maintain consistency with our previous publication. Following that, they were thoroughly rinsed in ultrapure water following the procedures from.^12^

### 2.2 Surface characterisation

Mass difference was measured using an analytical digital balance (±1 mg) by weighting each sample before and after treatment.

A field emission scanning electron microscope (FESEM, Hitachi SU3900, Tokyo, Japan) at 1–30 kV was used to investigate the surface topography of the treated samples.

A contact angle goniometer (Dataphysicals OCA 15 EC) was used to measure the surface wettability of the samples. A 2 µL droplet of distilled water was applied to each sample surface; 3 s after contact, a photograph was taken using a high-resolution camera for the analysis.

Surface roughness parameters (*R*_a_, *R*_q_ and *R*_z_) of untreated and treated Zn samples were measured using a portable surface roughness tester (Surftest SJ-201 series, Mitutoyo America Corporation, Tokyo, Japan) with a measuring range of 16 mm. A measuring speed of 0.25 mm s^−1^ and detector measuring force of 0.75 mN testing parameters were used. The instrument was equipped with a diamond-tipped stylus with a 60° tip angle and 2 µm tip radius. Roughness measurements were performed with a cut-off length of 0.8 mm and a scan length of 4 mm. Three specimens from each sample group were tested, and three measurements were taken on each specimen (*n* = 9 per group). The mean values and standard deviations were then calculated.

Surface hardness of was evaluated using a Micro Vickers Hardness tester (MMT7, Matsuzawa, Japan) under a 25 gf (equivalent to 0.245 N) compressive load for 15 s.

### 2.3 Electrochemical measurements

To evaluate the degradation behaviour in different media, electrochemical tests were carried out using a potentiostat/galvanostat (PARSTAT 2273, AMETEK) in three different electrolytic solutions with the pH adjusted to 7.4 ± 0.2. Details about the compositions of the solutions are listed in Table 1. Long-term immersion tests were conducted to evaluate the degradation behaviour of untreated and chemically treated Zn samples in three simulated physiological solutions (HBSS, 0.85 wt% NaCl, and 10 mM Tris–HCl). Each sample was immersed individually in a sealed polypropylene container containing 50 mL of solution, corresponding to a solution volume-to-exposed surface area ratio of approximately 20 mL cm^−2^, which is commonly recommended for corrosion testing of biodegradable metals. All immersion tests were performed at 37 ± 0.5 °C in a temperature-controlled incubator. The containers were kept under static conditions without mechanical stirring to simulate a relatively quiescent physiological environment. The immersion solutions were renewed every 3 days to maintain stable ionic composition and prevent excessive accumulation of corrosion products. The pH of the immersion solutions was monitored periodically using a calibrated pH meter to evaluate changes during degradation. All tests were conducted under ambient laboratory atmosphere, without additional gas control. After 28 days of immersion, the samples were removed, gently rinsed with deionized water, dried in ambient air, and subsequently characterized by SEM, EDS and optical microscopy to evaluate corrosion morphology and surface products. The immersion conditions were selected according to commonly used practices for evaluating corrosion behaviour of biodegradable metals in simulated physiological environments.

**Table 1:** Reagents employed for the preparation of the three physiological solutions (g L^−1^)

Solution	HBSS	0.85 wt% NaCl	10 mM Tris–HCl
NaCl	8.000	0.850	—
NaHCO_3_	0.350	—	—
KCl	0.300	—	—
KH_2_PO_4_	0.120	—	—
CaCl_2_	0.185	—	—
Na_2_HPO_4_	0.047	—	—
MgSO_4_·2H_2_O	0.097	—	—
Tris	—	—	6.118

All electrochemical tests were performed at 37 ± 0.5 °C. A platinum electrode (10 × 15 × 0.2 mm platinum plate), and the sample were connected to the reference electrode (saturated calomel electrode, SCE), counter electrode, and working electrode, respectively. The samples were mounted in a non-conductive epoxy holder, leaving an exposed circular working area of 0.25 cm^2^. Care was taken to ensure a tight seal between the sample and holder to prevent crevice corrosion at the sample edges. Prior to each electrochemical test, the exposed surface was gently rinsed with ethanol and deionized water and allowed to stabilize in the electrolyte until the open circuit potential reached a steady state. These electrodes were used for the electrochemical tests of open circuit potentials (OCP), electrochemical impedance spectroscopy (EIS), and potentiodynamic polarisation (PDP). The EIS tests were carried out after reaching a steady state open circuit for up to 4000 s, with a voltage perturbation amplitude of 10 mV and a frequency range from 10^5^ Hz to 10^−2^ Hz. The potential polarisation tests were carried out with a scanning range of −300 to +600 mV *versus* OCP at a scan rate of 1.0 mV s^−1^. At least three specimens were tested for each group. The EIS behaviour of the materials was modeled and analysed using Cview. The rate of degradation was evaluated by Tafel extrapolation of the measured cathodic potentiodynamic polarisation curves of the specimens. Corrosion potential (*E*_corr_) and corrosion current density (*i*_corr_) were determined from the polarisation curves.^14^ The degradation rate was calculated using an equation based on the ASTM G102 standard:CR = 3.27 × 10^−3^ × *i*_corr_ × EW/*ρ*where *ρ* is the density of the sample and EW is the equivalent weight. Based on the degradation rate, the lifetime of the samples can be calculated. Meanwhile, the corrosion morphology and surface products were also characterized using FE-SEM.

### 2.4 Statistical analysis

All quantitative data are presented as mean ± standard deviation (SD). Unless otherwise specified, all measurements were performed using at least three independent samples (*n* ≥ 3). Electrochemical tests were performed with five replicates (*n* = 5). Statistical analyses were conducted using one-way analysis of variance (ANOVA) to evaluate differences among multiple groups. When statistically significant differences were identified, Tukey's post hoc multiple comparison test was applised to determine pairwise significance. A value of *p* < 0.05 was considered statistically significant. Data analysis and graphical representation were carried out using GraphPad Prism software (GraphPad Software, USA).

## 3. Results

### 3.1 Surface characterisation before and after chemical treatment

**Fig. 1:**
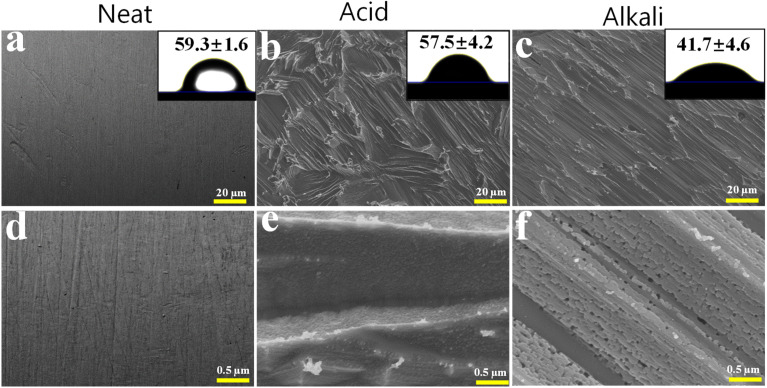
FESEM morphological observation at two different magnifications of (a and d) neat (untreated); (b and e) acid-treated and (c and f) alkali-treated Zn samples. WCA 2D picture and its value is shown as inset of panels a, b and c.

Our group recently published the detailed etching protocol, characterisation, and mechanism of acid-etched samples followed by alkaline treatment;^13^ in the present study, this sequential acid + NaOH condition is referred to as ‘alkali-treated’ for consistency with that previous work. The surface morphology of the Zn samples was observed by SEM to compare the changes before and after acid and alkaline chemical treatments (Fig. 1). On the untreated Zn surface, directional scratches caused by the grinding and polishing processes were clearly observed (Fig. 1a and d). After acid treatment, these scratches were completely removed, leaving visible grooves and textures on the surface (Fig. 1b and e). The subsequent alkali treatment builds on this foundation by introducing sub-micron decorative structures superimposed on the microscopically rough surface created by the acid treatment, resulting in a hierarchical micro-nano surface topography (Fig. 1c and f).

Surface roughness and wettability analyses showed that the acid-treated Zn-based materials exhibited higher surface roughness compared to the untreated Zn-based samples, with a significant increase in *R*_a_ (arithmetical mean roughness), *R*_q_ (root mean square roughness), and *R*_z_ (average maximum height) values (Table 2), confirming the pronounced micro-scale topography generated by the etching process. After the subsequent alkali treatment, the roughness values remained at a similarly elevated level (with only minor variations among *R*_a_, *R*_q_ and *R*_z_) and were still markedly higher than those of untreated Zn, indicating that the formation of additional nanoscale features did not smooth the surface. In addition, the wettability of the materials was evaluated by determining the contact angle of water droplets on the sample surface. Acid treatment resulted in a measurable decrease in contact angle compared with untreated Zn (Table 2). Statistical analysis confirmed that the differences between groups were significant (*p* < 0.05). In dental and orthopedic implant applications, the mechanical stability of the surface is critical, as any exfoliation or release of the nanoscale structures may cause a toxic reaction in localized cells or inflammatory responses.^15^ The alkali-treated Zn samples exhibited higher hardness than the untreated and solely acid-treated samples (Table 2), suggesting that the chemically formed surface layer possesses improved mechanical integrity and is less prone to mechanical damage under service conditions.

**Table 2:** Characterization of untreated and treated Zn samples

	Weight loss^a^ (%)	Surface roughness (µm)			Hardness (Hv)
		*R*_a_	*R*_q_	*R*_z_	
Neat Zn	—	0.7 ± 0.2	1.1 ± 0.3	5.8 ± 1.1	30.4 ± 1.1
Acid	1.7 ± 0.3	2 ± 0.2	2.5 ± 0.3	13.9 ± 1.9	39.1 ± 5.6
Alkali	0.25 ± 0.1	1.8 ± 0.4	2.3 ± 0.6	14.4 ± 3.7	42.3 ± 3.4

a Weight loss of the pure Zn samples after acid and alkali treatments.

### 3.2 Surface characterization after immersion test

**Fig. 2:**
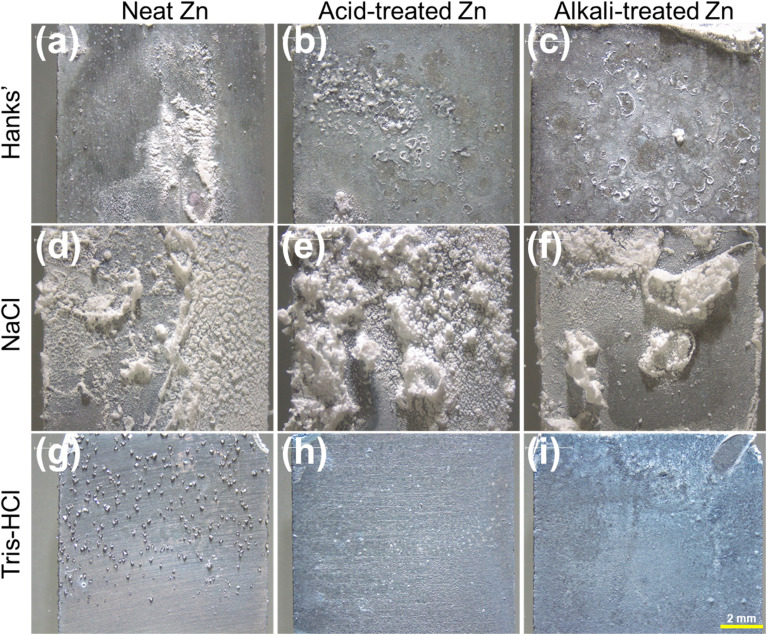
3D optical images of (a, d and g) neat (untreated), (b, e and h) acid-treated and (c, f and i) alkali-treated Zn samples after immersion for 28 days at 37 °C in the three simulated physiological solutions.

The surface morphology of the Zn samples after 28 days of immersion in HBSS, NaCl, and Tris–HCl was characterized by optical microscopy (Fig. 2). The surface of the untreated Zn samples exposed to HBSS was partially covered by white precipitates in the form of agglomerates, while directional scratches left by the polishing process could still be seen in the areas not covered by these precipitates (Fig. 2a).^16^ The samples chemically treated with acid and alkali were uniformly scattered with white corrosion products, indicating a more homogeneous corrosion process (Fig. 2b and c).

The samples immersed in NaCl solution, both untreated and chemical-treated Zn samples, showed extensive and thicker corrosion layer formation, with the surface of the acid-treated Zn samples almost completely covered by a dense, compact layer of corrosion products (Fig. 2e). The alkali-treated samples also showed substantial corrosion product formation (Fig. 2f), although some underlying surface features were still discernible. In contrast, the surface of the samples placed in Tris–HCl solution showed no signs of severe corrosion (Fig. 2g–i). The untreated Zn samples had clusters of precipitates scattered on the surface (Fig. 2g). Acid-treated Zn displayed a more continuous but relatively thin layer of deposits (Fig. 2h). The alkali-treated Zn samples (Fig. 2i) retained a largely intact surface appearance with limited and finely distributed corrosion products, indicating a more moderate and uniform degradation behaviour in Tris–HCl compared with HBSS and NaCl.

### 3.3 Microstructural analysis of surface corrosion products

**Fig. 3:**
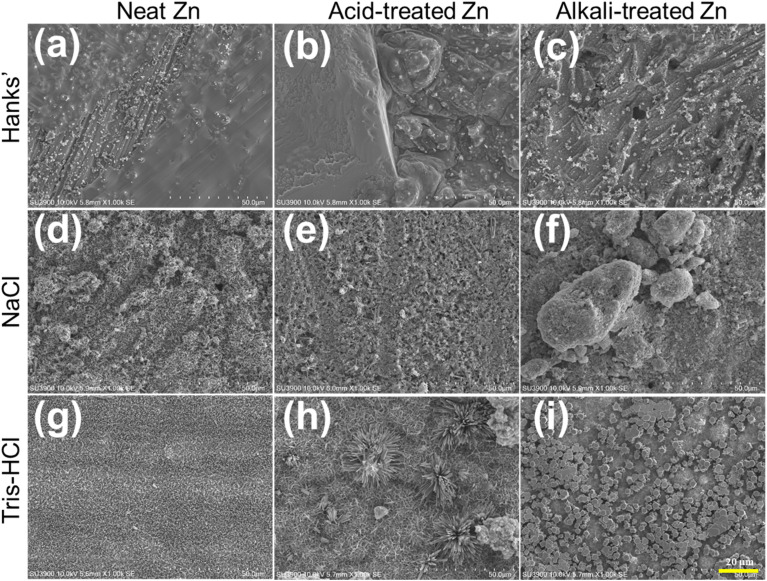
The SEM shows the surface morphology of (a, d and g) neat (untreated), (b, e and h) acid-treated and (c, f and i) alkali-treated Zn samples after immersion for 28 days at 37 °C in the three simulated physiological solutions.

Fig. 3 showed the SEM morphology of corrosion products on Zn samples after 28 days of immersion in HBSS, NaCl, and Tris–HCl solutions. In HBSS, the untreated Zn samples only formed a thin and discontinuous corrosion layer on the surface with granular corrosion products scattered on top, and scratches formed during polishing process were still visible (Fig. 3a). In contrast, the surface of the acid-treated Zn samples showed dense and coarse shingle-like corrosion products (Fig. 3b), while the surface of the alkali-treated samples showed a uniform distribution of crystalline fine-grained corrosion products (Fig. 3c). In NaCl solution, the surfaces of all samples were covered with a large number of multilamellar corrosion products, demonstrating the strong corrosive effect of NaCl on Zn (Fig. 3d–f). In particular, a unique pebble-like morphology was observed on the surface of alkali-treated Zn samples (Fig. 3f). The Zn samples exhibited unique corrosion patterns in Tris–HCl solution: the untreated samples formed a uniform layer of acicular corrosion products on the surface (Fig. 3g), while the acid-treated samples exhibited dispersed microflowering corrosion products (Fig. 3h), and the alkali treatment enhanced the enrichment of corrosion products on the surface to form a squashed sphere-like structure (Fig. 3i). These distinct morphologies indicate that chemical surface treatment governs not only the extent but also the spatial distribution and chemistry of corrosion products, suggesting fundamentally different degradation pathways in each medium.

### 3.4 Chemical composition analysis of surface corrosion products

By EDS analysis of the samples after immersion, Fig. S1 of SI shows the major elemental composition of the sample surface after immersion in different solutions for 28 days. Zn, oxygen (O), and carbon (C) were the major elements on the surface of the samples in all solutions, while the elements phosphorus (P) and calcium (Ca) were also detected in HBSS group. Acid treatment significantly reduced the amount of Zn on the surface of the samples compared to the untreated samples. The alkali-treated samples, although the Zn content recovered, was still lower than that of the untreated Zn samples. In particular, the alkali-treated Zn samples showed significantly more Ca/P precipitates formed on the surface of the samples in HBSS compared to the untreated Zn samples, and small amounts of Ca and P were also detected on specimens immersed in NaCl and Tris–HCl solutions. This is an important indicator for assessing the bioactivity associated with orthopedic applications. In particular, the alkali-treated samples showed a significantly greater ability to form Ca/P precipitates on the surface than the acid-treated samples. It should be noted that the present compositional analysis is based primarily on EDS measurements, which provide elemental information but do not directly resolve the crystalline phases of the corrosion products. It should be noted that the present compositional analysis is primarily based on EDS measurements, which provide elemental information but do not directly resolve the crystallographic phases of corrosion products. While the presence of Zn, O, Ca and P suggests the formation of zinc oxides/hydroxides and Ca/P-containing deposits in HBSS, the exact chemical phases (*e.g.*, carbonate, phosphate or mixed compounds) cannot be conclusively identified using EDS alone. Therefore, the interpretation of corrosion products in this study is based on the combined analysis of elemental distribution, surface morphology and electrochemical behaviour. Future work incorporating phase-sensitive techniques such as X-ray diffraction (XRD), X-ray photoelectron spectroscopy (XPS), Raman spectroscopy or FTIR would provide more definitive identification of the corrosion products formed on chemically treated Zn surfaces.^17^

### 3.5 Electrochemical performance evaluation

**Fig. 4:**
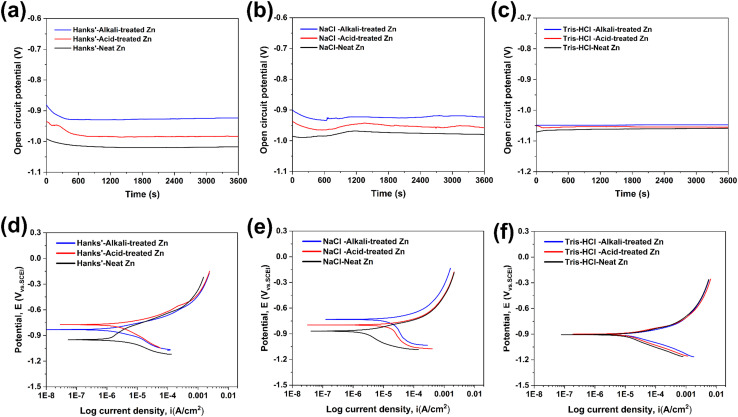
OCP (a–c) and (d–f) PDP curves for untreated and chemically treated Zn samples in HBSS, NaCl, and Tris–HCl solutions (*n* = 5).

The corrosion performance of studied Zn samples was investigated using transient electrochemical corrosion techniques over a short period of time. Fig. 4a–c shows the variation of the OCP with time during the immersion of Zn in three different corrosion solutions for 3600 s. The results show that the OCP of the acid-treated Zn samples increased compared to the untreated Zn in all the solutions tested, while the OCP of the alkali-treated Zn samples continued to increase, suggesting that the surface stability of the chemically treated Zn was improved.^11^ In particular, the initial decrease in OCP in HBSS and NaCl solutions may be related to the rupture of the surface film, whereas in Tris–HCl solution, the OCP of Zn shows a more stable trend with only slight fluctuations.

**Fig. 5:**
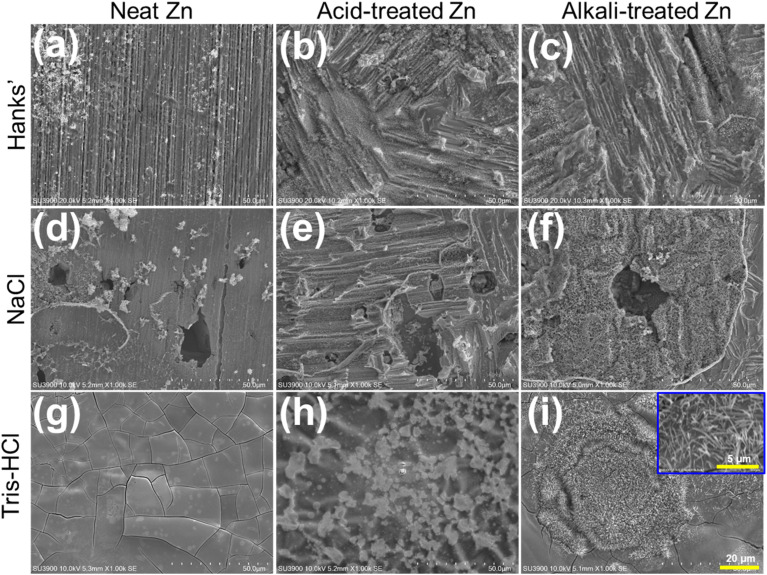
Illustrates the surface morphology of (a, d and g) neat (untreated) and (b, e and h) acid-treated and (c, f and i) alkali-treated Zn samples after an electrochemical corrosion test in the three simulated physiological solutions. Inset shows higher magnification image.

In addition, PDP tests were carried out to provide information on the thermodynamic and kinetic properties of the Zn degradation process. Fig. 4d–f shows the PDP curves of Zn samples in three solutions. The corrosion potentials (*E*_corr_) of the chemically treated Zn samples drifted in a positive direction in HBSS and NaCl solutions, with the acid-treated samples showing a slightly stronger cathodic polarisation tendency compared to the alkali-treated samples, indicating that the acid-etched Zn surface is more susceptible to corrosion than the alkali-treated surface in these media. It is noteworthy that, compared to untreated pure Zn, chemically treated Zn shows a different behaviour in the anodic part compared to the passivation-like narrow protected zone, with a higher cathodic dissolution rate. The SEM of the corrosion product morphology on the sample surface is shown in Fig. 5, and the parameters of OCP and PDP are summarized in Table 3. On the basis of the calculated corrosion rates (CR), in HBSS and NaCl the degradation rates followed the trend acid-treated Zn > alkali-treated Zn > neat Zn, showing that the chemical treatments accelerate corrosion in these media, with the acid-etched surface being the most reactive. In Tris–HCl, both chemically treated groups again exhibited higher CR values than neat Zn, and the alkali-treated specimens showed the highest corrosion rate. For all three corrosive media, the corrosion rate of Zn decreased in the order NaCl > Tris–HCl > HBSS.

**Table 3:** Open circuit potential, corrosion potential, and corrosion rate for electrochemical tested groups in HBSS, NaCl, and Tris–HCl solutions

Material	Open circuit potential (V, *vs.* SCE)	Corrosion current density, *i*_corr_ (µA cm^−2^)	Corrosion potential, *E*_corr_ (V, *vs.* SCE)	Corrosion rate, CR (mm per year)
HBSS′-neat Zn	−1.017 ± 0.1	0.857 ± 0.09	−1.002 ± 0.11	0.011 ± 0.005
HBSS′-acid-treated Zn	−0.982 ± 0.12	1.929 ± 0.3	−0.955 ± 0.9	0.059 ± 0.002
HBSS′-alkali-treated Zn	−1.010 ± 0.16	1.776 ± 0.16	−0.963 ± 0.14	0.034 ± 0.008
NaCl-neat Zn	−0.980 ± 0.05	2.890 ± 0.2	−1.054 ± 0.18	0.055 ± 0.002
NaCl-acid-treated Zn	−0.962 ± 0.08	15.234 ± 1.5	−1.065 ± 0.92	0.569 ± 0.07
NaCl-alkali-treated Zn	−0.974 ± 0.07	12.411 ± 1.3	−1.024 ± 0.14	0.381 ± 0.05
Tris–HCl-neat Zn	−1.059 ± 0.05	42.541 ± 3.5	−1.133 ± 0.17	0.126 ± 0.04
Tris–HCl-acid-treated Zn	−1.052 ± 0.1	58.464 ± 4.8	−1.126 ± 0.05	0.342 ± 0.072
Tris–HCl-alkali-treated Zn	−1.070 ± 0.13	105.940 ± 12.2	−1.137 ± 0.07	0.390 ± 0.05

### 3.6 Electrochemical impedance spectroscopy (EIS) analysis

**Fig. 6:**
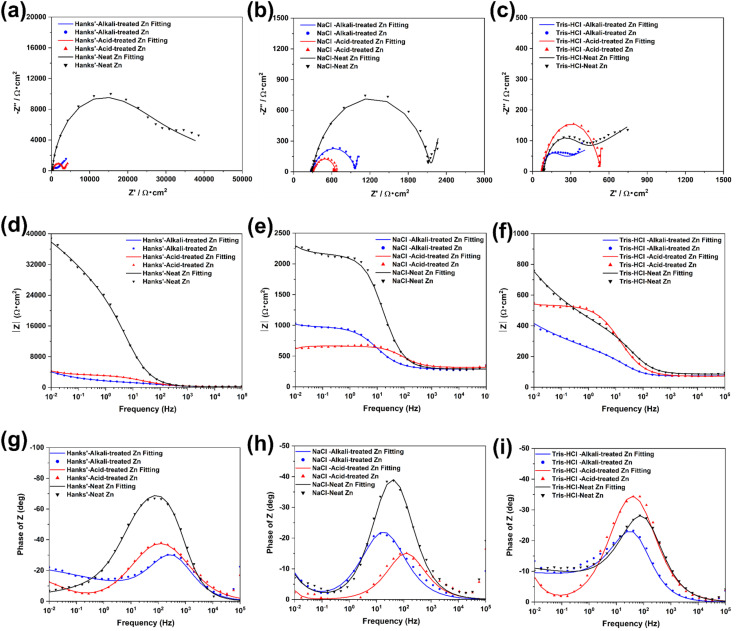
Representative EIS results of untreated and chemically treated Zn samples immersed in HBSS, NaCl, and Tris–HCl solutions: (a–c) Nyquist plots, (d–f) Bode plots, and (g–i) phase angle plots (*n* = 5). The measured real *Z*′ and imaginary *Z*″ parts of the impedance were normalized to the exposed geometric surface area of the working electrode (Ω cm^2^) to ensure an accurate comparison of intrinsic corrosion resistance across all samples.

The selected equivalent circuit model has been widely used to describe corrosion processes of biodegradable metals in physiological environments, where the high-frequency loop corresponds to the double-layer response and the low-frequency loop represents the corrosion product layer. EIS reveals the electrochemical kinetic behaviour of chemically treated Zn samples under different corrosion environments using Nyquist (Fig. 6a–c), Bode (Fig. 6d–f), and phase angle plots (Fig. 6g–i). The Nyquist plots revealed two significant time constants corresponding to the charge transfer process associated with the electrochemical double layer in the high-frequency region and the capacitive loops present in the corrosion product layer in the low-frequency region.^18^ By comparing the Nyquist plots of the different samples in the three solutions, the untreated Zn shows the largest capacitance loops, implying that it has the lowest corrosion rate under these conditions. The acid-treated Zn samples showed a decrease in the diameter of the Nyquist plots, while the alkali-treated samples showed a slight increase, particularly in HBSS, which had the largest size. This result was consistent with the corrosion rate trend observed in the potentiodynamic polarisation measurements.

To further interpret the EIS data, an electrical equivalent circuit (EEC) with a quadratic time constant was fitted. The schematic of the circuit is shown in Fig. S2, and the results of the fitting are summarised in Table 4. In this circuit model, *R*_s_ represents the solution resistance, *R*_f_ represents the film resistance, and *R*_ct_ denotes the charge transfer resistance, where the magnitude of *R*_f_ is a direct reflection of the corrosion resistance of the material. In addition, a constant phase element (CPE) is used in the equivalent circuit instead of a conventional capacitor to describe the surface dispersion effect observed in EIS measurements.^19^ The behaviour of the CPE can be caused by a number of factors, including the distributed nature of the surface reaction, surface roughness, porosity, and uneven distribution of currents and potentials due to the shape of the electrodes.

**Table 4:** Average values of EIS-derived parameters obtained from equivalent circuit fitting for untreated and chemically treated Zn samples after immersion in HBSS, NaCl, and Tris–HCl solutions (mean ± SD, *n* = 5)

Medium	Sample	*R*_s_ (Ω cm^2^)	*R*_f_ (Ω cm^2^)	*R*_ct_ (Ω cm^2^)	CPE-T (µF cm^−2^ s^*n*−1^)	*n*_f_
HBSS	Neat Zn	13.1 ± 1.7	1785 ± 232	895 ± 140	82 ± 10	0.87 ± 0.02
	Acid-treated Zn	12.9 ± 1.8	690 ± 98	422 ± 78	128 ± 18	0.81 ± 0.02
	Alkali-treated Zn	13.1 ± 1.7	1390 ± 175	705 ± 103	98 ± 13	0.85 ± 0.02
NaCl	Neat Zn	15.1 ± 1.7	977 ± 138	535 ± 92	145 ± 19	0.78 ± 0.03
	Acid-treated Zn	14.9 ± 2.3	396 ± 72	218 ± 45	184 ± 21	0.77 ± 0.03
	Alkali-treated Zn	13.5 ± 1.8	598 ± 85	362 ± 68	158 ± 21	0.81 ± 0.03
Tris–HCl	Neat Zn	17.1 ± 1.9	801 ± 125	412 ± 82	178 ± 25	0.80 ± 0.03
	Acid-treated Zn	16.8 ± 2.7	385 ± 62	178 ± 31	238 ± 25	0.74 ± 0.03
	Alkali-treated Zn	16.9 ± 2.6	322 ± 58	158 ± 33	271 ± 28	0.69 ± 0.04

The impedance of the CPE can be given by the following equation:*Z*_CPE_ = [*T*(*jω*)^*n*^]^−1^where *Z*_CPE_ is the CPE impedance (Ω cm^2^), *j* is the imaginary unit (−1)^0.5^, *ω* is the radial frequency of AC signal (*ω* = 2π*f*, *f* is the frequency), *T* is the capacitive effect of the interface (Ω cm^−2^ s^*n*^), and the range of number *n* is 0–1, which is represented as an ideal capacitor when the value equal to 1.^20^

In this study, the polarisation resistance (*R*_p_) of untreated pure Zn was greater than that of chemically treated Zn samples. The low-frequency impedance modulus (*Z*) in the Bode plot is considered to be a key indicator of the corrosion resistance of a material, where a higher *Z* value indicates a lower corrosion rate. The untreated Zn had the highest *Z* value. In addition, the trends of the Bode and phase angle plots shown in Fig. 6d–i are consistent with the results of the Nyquist plots, further confirming the differences in corrosion resistance of the Zn samples under different treatment conditions. These observations indicate that the chemically modified Zn surface does not respond uniformly across physiological environments, but instead selectively activates distinct corrosion pathways depending on solution chemistry.

## 4. Discussions

### 4.1 Effect of chemical treatment on Zn surface morphology

Implant surface roughness plays a critical role in the formation of bone tissue at the bone-implant interface.^21^ The functional activity of cell contact with the biomaterial surface, and in particular the surface roughness, is critical in promoting cell adhesion and subsequent bone formation.^22^ Immediately after implantation, the surface of the biomaterial is covered with high concentrations of cell adhesion proteins such as fibronectin and vitronectin from blood serum proteins. These proteins provide attachment sites for osteoblast precursors, and the increased protein adsorption capacity promotes binding to the implant, which in turn accelerates intraosseous growth and improves implant stability.

In this study, the rough and stable surface structure formed by acid and alkali treatment of Zn surfaces was investigated. Our previous study showed that etched Zn-based grafts had enhanced surface protein adsorption and cell adhesion capacity compared to untreated pure Zn.^11^ The present surface characterization showed that acid-etched Zn possessed higher roughness and improved wettability, and the surface affinity was further enhanced by alkali treatment. As previously reported, Zn ions can undergo an *in situ* crystallization–dissolution–precipitation process by dehydration and intra-atomic rearrangement in concentrated NaOH solution, which induces the surface nanorod-like oxidation products to grow in a specific direction and form structures with increasing length, high aspect ratio, and tapered ends, which are important for enhancing the bioactivity of the implant surfaces and facilitating the tight bonding of bone tissues.^23^

Chemical pre-treatment not only changes the morphology of the implant surface, but also plays a critical role in adjusting the chemical properties and wettability of the surface. The wettability of implant surfaces is a key factor influencing blood/plasma protein adhesion, cellular interactions, and bacterial attachment. Hydrophilic surfaces have been shown to promote the proliferation and attachment of osteoblasts and fibroblasts. Acid-treated implant surfaces have shown improved wettability. A study by Wenzel *et al.* found an inverse relationship between surface roughness and water contact angle, *i.e.* as surface roughness increased, wettability increased.^24^ After alkali treatment, the surface of the samples exhibited more hydrophilic behaviour, mainly due to the introduction of hydroxyl groups by the alkali treatment. The introduction of hydroxyl groups not only helps to increase the affinity of cells for the material, but Zhu *et al.* also demonstrated that it is an effective way to promote cell attachment.^25,26^ In addition, the nanoscale craters formed on the sample surface after alkali treatment also helped to improve surface wettability, while the nanoscale relief structures produced by the combined acid–alkali treatment effectively improved implant wettability.^27^

In addition, maintaining a certain mechanical strength to match human bone as closely as possible is a desirable goal in the process of surface modification of biomaterials. The alkali-treated samples showed improved mechanical strength, which may be related to the microscale roughening of the treated surfaces, which is a key factor in the implantation process and stability after long-term mechanical loading.^28^

### 4.2 Influence of chemical treatments on the corrosion performance of Zn

For biodegradable metallic materials, corrosion performance is considered one of the evaluation criteria and this performance is influenced by several factors, including the standard electrode potential of the metal itself, the surface geometry, the surface treatment, and the corrosive medium.^29^ In particular, for Zn-based implants, the chemical composition and microstructure of the surface coating are critical in determining the corrosion resistance and degradation behaviour of the implant in the biological environment.^30^ This study shows that it is possible to effectively control the morphology and structure of Zn surfaces by pre-treatment.

In recent years, Zn and its base alloys have received much attention in the biomedical field due to their ability to match the natural repair rate of the human body, as their corrosion rate is intermediate between that of magnesium and iron. However, the degradation rate of pure Zn is typically slow, with annual degradation rates of only a few tens of microns, far below the approximately 0.5 mm per year desired in orthopedic applications, which limits its use in certain application scenarios where long-term implant residues may impede bone tissue formation.^31^ Despite research efforts to tune the mechanical properties of pure Zn and improve its cytocompatibility by developing new alloys, the corrosion rates of these new alloys tend to be lower than those of unalloyed Zn. For example, the Zn-5Ge alloy developed by Tong *et al.* had a lower corrosion rate than pure Zn because the addition of elements changed the equilibrium electrode potential with pure Zn, creating a microscopic barrier, and this change also affected its mechanical properties.^32^ In the present study, both acid and alkali treatments increased the corrosion rate of Zn in all three media compared with untreated Zn, while the alkali-treated surface in HBSS maintained a relatively moderate and more uniform degradation. Previous studies have provided insight into the corrosion behaviour of pure Zn in a variety of corrosive media such as HBSS and NaCl solution, and this work is the first to report the corrosion properties of chemically treated Zn.^33^

#### 4.2.1 Corrosion behaviour of chemically treated Zn in HBSS

In HBSS, chemical treatment had a significant effect on the corrosion behaviour of Zn. The corrosion process of Zn involves two main partial reactions: the reduction of oxygen as the main cathodic reaction and the dissolution of Zn as the anodic reaction. Together these two reactions promote an increase in Zn^2+^ and OH^−^, contributing to the formation of Zn(OH)_2_, which is subsequently converted to the more stable ZnO.^34^ As OH^−^ continues to increase with pH, the Zn acid radical ion Zn(OH)_4_^2−^ is formed at the highly active cathodic site at a sufficiently high pH, while the depletion of OH^−^ causes the pH to decrease.

When compared after 28 days of immersion, the untreated Zn sample showed a higher polarisation resistance *R*_p_ due to the accumulation of insoluble salts forming a thicker layer of corrosion products. In contrast, the acid-etched Zn accelerated the corrosion process due to the increase in surface area, resulting in a lower *R*_p_ value, reflecting a weakened impedance response. In comparison, the alkali-treated Zn showed slightly improved corrosion resistance due to the formation of Ca–P salts from Ca^2+^ and HPO_4_^2−^ in HBSS and the dispersion and growth of phosphate and carbonate agglomerates on the Zn surface, which reduced its corrosion rate.^35^ This is supported by the increase in the Ca/P ratio observed by EDS analysis of the surface corrosion products. The lower Ca/P exhibited on the untreated Zn was due to the formation of a dense corrosion layer on the untreated Zn consisting mainly of ZnO and Zn(OH)_2_, demonstrating that ZnO, as a degradation product stable in physiological environments, with its lower surface energy and smoother surface, provides fewer nucleation sites and energy for Ca/P compounds, which in turn reduces the formation of Ca/P precipitates.^36^ The Ca/P ratio was also found to be higher on the untreated Zn than on the untreated Zn. P has been shown to be an important indicator of biological activity relevant to orthopedic applications.^12^ A study by Bowen *et al.* on the *in vivo* performance of pure Zn wires in mouse arteries showed a degradation rate of 0.012 mm per year during 1.5 months of exposure and the formation of a dense layer of corrosion products consisting mainly of Zn oxide and Zn carbonate after 4.5 months, with a calcium/phosphorus phase observed at the surface, further confirming the beneficial effect of Ca/P corrosion products.^37^ Considering the potential of Zn to promote osteoblast mineralization with anti-inflammatory properties, the increase in Ca/P corrosion products by convenient alkali treatment may further enhance the potential of Zn for medical applications such as orthopedic and cardiovascular stents. It should be noted that the present results primarily reveal a correlation between the enrichment of Ca/P phases on the alkali-treated Zn surface and the observed moderation and homogenisation of corrosion behaviour in HBSS. While the EDS analysis and surface morphology indicate enhanced Ca/P precipitation on the treated samples, the current study does not quantitatively isolate the direct causal contribution of these deposits to corrosion mitigation. Therefore, the role of Ca/P-rich layers should be interpreted as a contributing interfacial factor that may influence degradation behaviour rather than a definitively proven passivation mechanism. Future studies employing quantitative phase analysis, *in situ* monitoring of surface evolution, and controlled precipitation experiments would be valuable to further clarify the mechanistic relationship between Ca/P deposition and corrosion regulation in Zn-based biodegradable systems. Although the present analysis identifies Ca/P enrichment using EDS, further phase-resolved characterization such as XRD would be beneficial to directly confirm the crystallographic phases of the corrosion products and better quantify their role in corrosion regulation.

#### 4.2.2 Corrosion behaviour of chemically treated Zn in NaCl

The corrosion behaviour of Zn after 28 days of immersion in NaCl solution was different from that in HBSS. The appearance of white precipitates on the Zn surface indicates that NaCl is more corrosive to Zn samples.^38^ Cl^−^ is known to be a readily adsorbed anion capable of penetrating the intergranular boundaries of metal/oxide interfaces, leading to localized film breakdown and consequently pitting or passivation.^39^ In alkali-treated samples, a dense, porous oxide layer is formed on the surface, and this structure makes it easier for Cl^−^ to migrate to the film/metal interface and react with Zn to form chloride ions which are known to destabilize corrosion product layers and may contribute to localized corrosion behaviour.^40^ The continued growth of these complexes creates internal stresses at the interface due to volume expansion, leading to localized film failure. Pits are then formed at the initial Zn sites, ultimately accelerating the corrosion rate of chemically treated samples.

#### 4.2.3 Corrosion behaviour of chemically treated Zn in Tris–HCl

The pH of tissue fluids is extremely important for the normal functioning of proteins and cells, and significant changes in pH can disrupt protein structure and affect function.^41^ The pH in the human body is mainly maintained by natural buffer systems such as HCO^3−^, HPO_4_^2−^ and plasma proteins.^42^ To investigate the mechanism of Zn degradation *in vitro*, previous studies have introduced HEPES and Tris–HCl buffer systems. Liu *et al.* showed that placing Zn in both Tris–HCl and SBF solutions accelerated its corrosion rate and induced severe pitting in NaCl solutions.^38^ In particular, the effect of Tris–HCl as a single corrosion medium on the corrosion of Zn has not been fully investigated.

In the case of Tris–HCl as a corrosion medium, OH^−^ generated by oxygen reduction is rapidly neutralized and consumed by H^+^ in Tris–HCl, thereby depleting OH^−^ and accelerating the oxidation process of Zn.^43^ This process involves reactive reactions at the electrode/electrolyte interface involving adsorbed intermediates, leading to a decrease in the stability of the Zn surface, a shift of the corrosion potential in a negative direction, and a decrease in the polarisation resistance *R*_p_. The result is increased dissolution of Zn and propagation of localized corrosion over the entire surface. In the present study, untreated and chemically treated Zn samples were compared and similarities in the impedance response were found, suggesting that there may be strong interactions between Tris and dissolved cations or corrosion products leading to activation of the sample surface.

In addition, photocomplexation between Tris and Zn^2+^ may facilitate the transport of Zn^2+^ ions from the interface into the solution and further delay the formation of a solid film due to mass transport limitations.^44^ It was also found that the HCl component of the Tris–HCl buffer system introduced a significant amount of Cl^−^, which may further accelerate the pitting of Zn and increase the corrosion rate.^45^ This finding is consistent with previous studies showing that Tris–HCl can be an effective buffer medium for assessing the *in vitro* corrosion performance of Zn-based alloys. Future studies may need to focus on the corrosion performance of Zn over different time periods to gain a more complete understanding.

#### 4.2.4 Integrated analysis of corrosion media

Although *in vitro* immersion tests cannot fully simulate the complexity of *in vivo* environments, systematic variation of the test media is essential to analyze individual physicochemical factors that control Zn degradation. This is particularly important for biodegradable Zn-based implants, whose long-term performance depends on a subtle balance between corrosion rate, corrosion mode and the biological activity of corrosion products. Previous comparative studies have shown that the choice of media (*e.g.* HBSS, SBF, NaCl, Tris–buffered solutions) can lead to markedly different corrosion rates and product chemistries for Mg- or Zn-based alloys, sometimes even reversing the relative ranking of materials.^46,47^ From this perspective, the three media employed here represent distinct regions within a broader “corrosion space” relevant to Zn-based implants. HBSS is a Ca/P-containing medium that better mimics the inorganic composition of interstitial fluid in bone tissue and promotes the precipitation of Ca/P-rich surface layers.^48^ In contrast, NaCl solution deliberately isolates the aggressive effect of chloride ions in the absence of protective Ca/P precipitation. Numerous studies on pure Zn and Zn alloys have shown that Cl^−^ is a dominant driver of localised attack, leading to porous, ZnCl_2_-containing films and the initiation of pits and crevices. The strong acceleration of corrosion observed for pre-treated Zn in NaCl therefore provides a “stress test” of the stability of the chemically modified surface when Ca/P passivation is absent.^49^ Tris–HCl occupies a third, mechanistically distinct regime, where the buffer not only controls bulk pH but also actively participates in interfacial reactions. For Zn or Mg, Tris–HCl has been reported to suppress the build-up of protective hydroxide/oxide layers, shift the corrosion potential negatively and promote more generalised dissolution by continuously neutralising OH^−^ and forming soluble complexes with metal cations.^50^ Taken together, the combined use of HBSS, NaCl and Tris–HCl provides a structured way to separate Ca/P-mediated passivation effects, chloride-driven localised corrosion and buffer/complexation-driven activation and thus lays a more solid foundation for interpreting future *in vivo* degradation data of chemically treated Zn implants.

#### 4.2.5 Surface chemistry-controlled degradation pathway model

Based on the combined electrochemical results, surface morphology and elemental composition analysis, a conceptual degradation pathway model for pure Zn can be proposed to explain the environment-dependent corrosion behaviour observed in this study. In Ca/P-containing media (HBSS), the alkali-treated Zn surface promotes rapid nucleation of Ca/P-rich deposits may contribute to partial surface stabilisation and redistribute anodic sites, resulting in moderated and more homogeneous degradation. In Ca/P-containing media (HBSS), the alkali-treated Zn surface promoted greater Ca/P enrichment, and this coincided with more spatially homogeneous and relatively moderated degradation behaviour compared with the acid-treated condition. These observations suggest that Ca/P-containing deposits may contribute to partial surface stabilisation in this medium. However, the present results do not quantitatively establish a direct causal relationship between Ca/P deposition and corrosion mitigation, and this interpretation should therefore be considered a plausible association based on the combined morphological, compositional, and electrochemical findings. In contrast, in chloride-dominated NaCl solution, aggressive Cl^−^ ions penetrate the chemically modified surface layer, destabilizing corrosion products and promoting localized attack. In Tris–HCl, continuous buffer neutralization suppresses protective film formation and enhances Zn^2+^ dissolution through complexation effects, leading to accelerated general corrosion. This mechanistic separation highlights that degradation behavior of Zn cannot be predicted from corrosion rate alone, but must be interpreted in the context of environment-specific interfacial chemistry. To consolidate the experimental observations across different physiological environments, the dominant chemical species, surface responses, and corresponding degradation modes of acid–alkali treated Zn are summarized in Table 5.

**Table 5:** Environment-specific degradation pathways of acid–alkali treated Zn

Medium	Dominant species	Surface response	Degradation mode	Design implication
HBSS	Ca^2+^/PO_4_^3−^	Ca/P-rich surface deposition	More homogeneous	Consistent with possible partial surface stabilisation
NaCl	Cl^−^	Film destabilization	Localized attack	Highlights susceptibility to chloride-driven attack
Tris–HCl	Buffer complexes	Enhanced dissolution	Accelerated general corrosion	Useful for accelerated screening and worst-case dissolution assessment

In particular, Ca/P-containing media were associated with partial surface stabilisation and more uniform degradation, whereas chloride- and buffer-dominated environments favored film destabilisation or enhanced dissolution, respectively.

This synthesis highlights that the same surface modification can activate fundamentally different degradation pathways depending on the surrounding solution chemistry. The comparison clearly demonstrates that degradation behavior of chemically treated Zn is governed by environment-specific interfacial chemistry rather than corrosion rate alone. Ca/P-containing media promote partial surface stabilization and more uniform degradation, whereas chloride- and buffer-dominated environments favor film destabilization or enhanced dissolution, respectively. It should be emphasized that the proposed degradation pathways are inferred from the combined electrochemical behaviour, morphology and elemental composition of corrosion products. While these observations provide consistent evidence for environment-dependent degradation behaviour, definitive phase identification of corrosion products would require complementary techniques such as XRD or XPS. These environment-dependent degradation pathways establish surface chemistry as an effective design parameter for programming Zn degradation behavior, providing guidance for selecting appropriate physiological testing environments and implant-specific applications.

### 4.3 Scope and boundary conditions of the present study

The present study was designed to systematically elucidate how simple acid–alkali surface chemistry influences the early-stage degradation pathways of pure Zn in distinct physiological environments. Accordingly, the experimental scope was intentionally focused on short-to-intermediate immersion periods and electrochemical characterization in well-defined simulated solutions, enabling mechanistic separation of Ca/P-mediated, chloride-driven, and buffer-controlled corrosion behaviors.

While long-term *in vivo* degradation, mechanical loading effects, and biological responses are essential for full translational assessment, these aspects were beyond the scope of the present work. Importantly, the insights provided here establish a fundamental framework for predicting and programming Zn degradation behavior through surface chemistry, which can directly inform future studies integrating mechanical performance, biological interactions, and implant-specific design requirements.

## 5. Conclusion

This work demonstrates that simple acid–alkali surface modification can significantly influence the degradation behaviour of pure zinc in different physiological environments. By systematically comparing corrosion behaviour in HBSS, NaCl, and Tris–HCl solutions, the results indicate that surface chemistry interacts strongly with solution composition, leading to environment-dependent degradation responses. Notably, alkali-treated Zn in HBSS showed greater Ca/P surface enrichment together with more homogeneous degradation features, suggesting a possible contribution of Ca/P-containing deposits to partial surface stabilisation under Ca/P-containing conditions. The combined electrochemical analysis, surface morphology observations, and elemental composition data suggest that the degradation pathways of chemically treated Zn are strongly influenced by interfacial reactions occurring in different solution chemistries. While the present results provide consistent evidence for these solution-dependent corrosion behaviours, further phase-sensitive characterization techniques would be valuable for fully resolving the composition of corrosion products and confirming the mechanistic pathways proposed. Overall, this study highlights the importance of considering solution chemistry when evaluating the degradation performance of biodegradable Zn and suggests that simple chemical surface treatments may offer a practical strategy for tailoring environment-specific degradation behaviour of Zn-based implants.

## Conflicts of interest

The authors declare no conflicts of interest.

## Supplementary Material

RA-016-D6RA00345A-s001

## Data Availability

The data supporting this study are available from the corresponding author upon reasonable request. Supplementary information (SI): SEM-EDX profiles and element composition of neat (untreated) and chemically treated Zn samples after immersion for 28 days at 37 °C, and the equivalent electrical circuit model. See DOI: https://doi.org/10.1039/d6ra00345a.
