# Supplementary material for: Environment-dependent degradation pathways of pure zinc induced by acid–alkali surface chemistry
Source: RSC Adv. 2026 Apr 21;16(22):20433–47. doi: 10.1039/d6ra00345a (PMC13096885; doi:10.1039/d6ra00345a)
Supplement: RA-016-D6RA00345A-s001 [file RA-016-D6RA00345A-s001.pdf]

Supplementary Information (SI) for RSC Advances.
This journal is © The Royal Society of Chemistry 2026

Supporting Information

For

Environment-dependent degradation pathways of pure zinc induced by acid–alkali surface chemistry

Abdalla Abdal-hay[1*], Enmao Xiang[1], Yahia Ali[2], Jiaqi Xu, Yu Kyoung Kim[4]

[1]Centre for Orofacial Regeneration, Reconstruction and Rehabilitation (COR3), School of Dentistry, The University of Queensland, Brisbane 4006, Australia;

[2]Centre for Advanced Materials Processing and Manufacturing (AMPAM), School of Mechanical and Mining Engineering, The University of Queensland, Brisbane, Qld, 4072, Australia[3]The Conversationalist Club & Department of Dental Digitalization, School of Stomatology, Shandong First Medical University, Jinan 250117, Shandong, China.

[4]Chonbuk National University, Institute of Oral Bioscience and School of Dentistry, Jeon Ju, South Korea;

Corresponding author: Email: abdalla.ali@uq.edu.au

Keywords: Biodegradable zinc; Bone regeneration; Degradation behaviour; Simulated physiological solutions; Surface modification

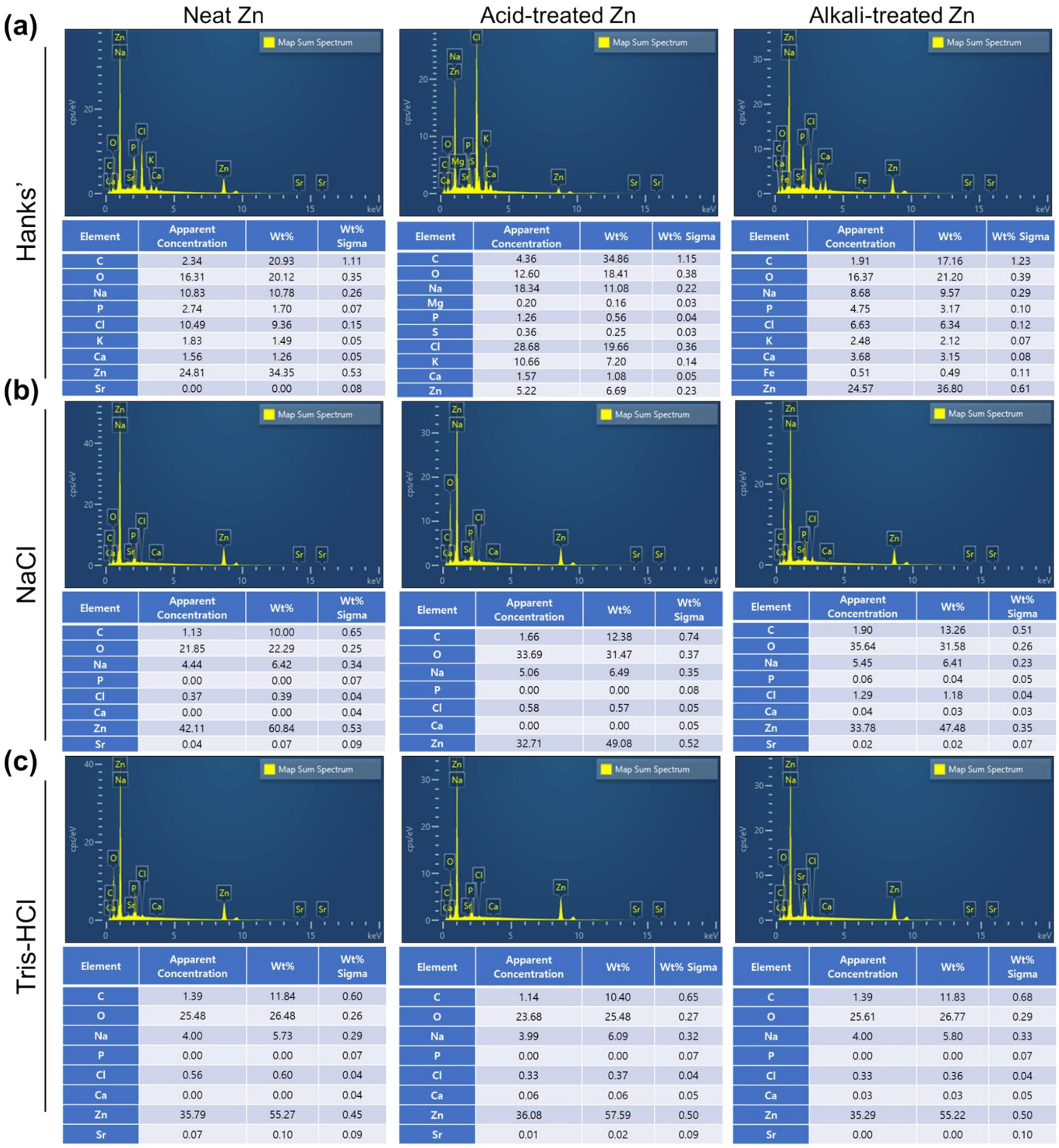

(a) Hanks' — Neat Zn

Element	Apparent Concentration	Wt%	Wt% Sigma
C	2.34	20.93	1.11
O	16.31	20.12	0.35
Na	10.83	10.78	0.26
P	2.74	1.70	0.07
Cl	10.49	9.36	0.15
K	1.83	1.49	0.05
Ca	1.56	1.26	0.05
Zn	24.81	34.35	0.53
Sr	0.00	0.00	0.08

(a) Hanks' — Acid-treated Zn

Element	Apparent Concentration	Wt%	Wt% Sigma
C	4.36	34.86	1.15
O	12.60	18.41	0.38
Na	18.34	11.08	0.22
Mg	0.20	0.16	0.03
P	1.26	0.56	0.04
S	0.36	0.25	0.03
Cl	28.68	19.66	0.36
K	10.66	7.20	0.14
Ca	1.57	1.08	0.05
Zn	5.22	6.69	0.23

(a) Hanks' — Alkali-treated Zn

Element	Apparent Concentration	Wt%	Wt% Sigma
C	1.91	17.16	1.23
O	16.37	21.20	0.39
Na	8.68	9.57	0.29
P	4.75	3.17	0.10
Cl	6.63	6.34	0.12
K	2.48	2.12	0.07
Ca	3.68	3.15	0.08
Fe	0.51	0.49	0.11
Zn	24.57	36.80	0.61

(b) NaCl — Neat Zn

Element	Apparent Concentration	Wt%	Wt% Sigma
C	1.13	10.00	0.65
O	21.85	22.29	0.25
Na	4.44	6.42	0.34
P	0.00	0.00	0.07
Cl	0.37	0.39	0.04
Ca	0.00	0.00	0.04
Zn	42.11	60.84	0.53
Sr	0.04	0.07	0.09

(b) NaCl — Acid-treated Zn

Element	Apparent Concentration	Wt%	Wt% Sigma
C	1.66	12.38	0.74
O	33.69	31.47	0.37
Na	5.06	6.49	0.35
P	0.00	0.00	0.08
Cl	0.58	0.57	0.05
Ca	0.00	0.00	0.05
Zn	32.71	49.08	0.52

(b) NaCl — Alkali-treated Zn

Element	Apparent Concentration	Wt%	Wt% Sigma
C	1.90	13.26	0.51
O	35.64	31.58	0.26
Na	5.45	6.41	0.23
P	0.06	0.04	0.05
Cl	1.29	1.18	0.04
Ca	0.04	0.03	0.03
Zn	33.78	47.48	0.35
Sr	0.02	0.02	0.07

(c) Tris-HCl — Neat Zn

Element	Apparent Concentration	Wt%	Wt% Sigma
C	1.39	11.84	0.60
O	25.48	26.48	0.26
Na	4.00	5.73	0.29
P	0.00	0.00	0.07
Cl	0.56	0.60	0.04
Ca	0.00	0.00	0.04
Zn	35.79	55.27	0.45
Sr	0.07	0.10	0.09

(c) Tris-HCl — Acid-treated Zn

Element	Apparent Concentration	Wt%	Wt% Sigma
C	1.14	10.40	0.65
O	23.68	25.48	0.27
Na	3.99	6.09	0.32
P	0.00	0.00	0.07
Cl	0.33	0.37	0.04
Ca	0.06	0.06	0.05
Zn	36.08	57.59	0.50
Sr	0.01	0.02	0.09

(c) Tris-HCl — Alkali-treated Zn

Element	Apparent Concentration	Wt%	Wt% Sigma
C	1.39	11.83	0.68
O	25.61	26.77	0.29
Na	4.00	5.80	0.33
P	0.00	0.00	0.07
Cl	0.33	0.36	0.04
Ca	0.03	0.03	0.05
Zn	35.29	55.22	0.50
Sr	0.00	0.00	0.10

Fig. S1. SEM-EDX profiles and element composition of neat (untreated) and chemically treated Zn samples after immersion for 28 days at 37°C in the (a) HBSS, (b) NaCl, (c) Tris-HCl.

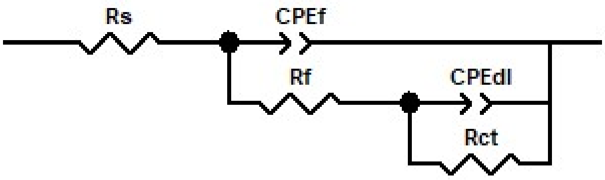

Figure S2. The equivalent electrical circuit model.
